# Quality of Systematic Reviews with Network Meta-Analyses on JAK Inhibitors in the Treatment of Rheumatoid Arthritis: Application of the AMSTAR 2 Scale

**DOI:** 10.3390/jcm15020725

**Published:** 2026-01-15

**Authors:** Bruna Ramalho, Ana Penedones, Diogo Mendes, Carlos Alves

**Affiliations:** 1 Laboratory of Social Pharmacy and Public Health, Faculty of Pharmacy, University of Coimbra, 3000-548 Coimbra, Portugal; brunaramalhobruna@hotmail.com (B.R.); apenedones@ff.uc.pt (A.P.); dmendes@ff.uc.pt (D.M.); 2 Clevidence, 2740-122 Oeiras, Portugal

**Keywords:** rheumatoid arthritis, JAK inhibitors, network meta-analysis, systematic reviews, AMSTAR 2, methodological quality, evidence-based medicine

## Abstract

**Background/Objective:** Systematic reviews (SRs) with network meta-analysis (NMA) support evidence-based decision-making by enabling both direct and indirect comparisons across multiple interventions. Given the expanding use of Janus kinase (JAK) inhibitors in rheumatoid arthritis (RA), the methodological rigor of SRs with NMA is essential for trustworthy conclusions. This study is aimed at evaluating the methodological quality of SRs with NMA assessing the efficacy and/or safety of JAK inhibitors in RA. **Methods:** PubMed and Embase were searched for full-text SRs with NMAs evaluating JAK inhibitors as a therapeutic class in RA. Eligible publications were English-language articles reporting efficacy and/or safety outcomes. Narrative reviews, letters, duplicates, reviews focused on a single JAK inhibitor, and reviews without quantitative synthesis were excluded. Three independent reviewers assessed methodological quality using AMSTAR 2. Descriptive statistics were used to summarize findings. **Results:** Of the 222 records identified, 18 SRs with NMA met the inclusion criteria: 5 focused on efficacy, 5 on safety, and 8 assessed both. The most consistently fulfilled AMSTAR 2 items were a clearly defined PICO question (100%), duplicate study selection (100%), and reporting of conflicts of interest (100%). Common shortcomings included lack of protocol registration (44%), incomplete reporting of the search strategy (39%), and absence of publication bias assessment (50%). Risk-of-bias assessment varied by review focus: all safety reviews complied (100%), compared with 20% of efficacy reviews and 37% of mixed reviews. **Conclusions:** Most SRs with NMA of JAK inhibitors in RA present relevant methodological limitations, particularly in protocol registration, search reporting, and risk-of-bias assessment. Methodological standards were generally higher in safety-focused reviews, underscoring the need for more consistent and rigorous conduct and reporting, especially in efficacy and mixed reviews, to strengthen confidence in NMA-derived conclusions.

## 1. Introduction

Rheumatoid arthritis (RA) is a chronic autoimmune condition marked by systemic inflammation that primarily affects synovial joints [1,2]. The disease typically leads to persistent joint inflammation, progressive destruction of cartilage and bone, and may also involve extra articular organs, such as the lungs, heart, and eyes [3]. Its clinical consequences include chronic pain, reduced mobility, and functional disability, all of which contribute to a substantial socioeconomic burden through increased healthcare utilization and decreased work productivity [3,4]. RA affects an estimated 0.5% to 1% of the global population, with a higher prevalence among women and a peak onset between 40 and 60 years of age [2]. The progressive nature of the disease, coupled with potential delays in diagnosis and intervention, continues to challenge effective long-term disease management. Therapeutic strategies for RA have advanced significantly, aiming to achieve disease remission or maintain low disease activity. Disease-modifying antirheumatic drugs (DMARDs) form the backbone of RA treatment and are categorized into conventional synthetic (csDMARDs), biological (bDMARDs), and targeted synthetic (tsDMARDs) agents [1]. Methotrexate remains the cornerstone of initial treatment; however, patients who fail to respond adequately may require the addition of either a bDMARD or a tsDMARD [2]. Among the tsDMARDs, Janus kinase (JAK) inhibitors have emerged as a relevant class due to their targeted mechanism of action on the JAK/STAT signaling pathway, which regulates key cytokines involved in RA pathophysiology [5]. These agents have demonstrated comparable or superior efficacy to bDMARDs in clinical trials and offer practical benefits, such as oral administration and rapid symptom control. Nevertheless, safety concerns, particularly infections, thromboembolic events, and potential cardiovascular and oncologic risks, have led regulatory bodies to issue updated recommendations for their use [6,7]. SRs play a pivotal role in evidence-based medicine, enabling the synthesis of findings from multiple studies to inform clinical and policy decisions. When supported by meta-analyses, SRs enhance the statistical power and precision of estimated treatment effects. Traditional meta-analyses, however, are limited to head-to-head comparisons. In contrast, network meta-analysis (NMA) allows for the comparison of multiple interventions simultaneously by combining both direct and indirect evidence across a network of trials [8,9]. In rheumatology, NMAs have become increasingly common in evaluating therapeutic options, including JAK inhibitors, and are often used to guide clinical guidelines and regulatory decisions [10,11]. Given their complexity, the validity of NMAs heavily depends on the methodological rigour of the underlying systematic reviews (SRs). Inconsistent reporting, absence of predefined protocols, and flawed risk of bias assessments can significantly affect the credibility of findings. These methodological inconsistencies raise particular concern when NMAs are used to underpin treatment recommendations. The AMSTAR 2 tool (A Measurement Tool to Assess Systematic Reviews) was developed to critically appraise SRs, whether they include meta-analyses and regardless of whether the primary studies are randomized or non-randomized [12]. This 16-item tool enables the identification of major methodological flaws and promotes the transparent and responsible use of published evidence. Considering the expanding role of JAK inhibitors in RA and the growing number of SRs with NMAs assessing their efficacy and safety, a structured evaluation of the methodological quality of these reviews is warranted. The present study aimed to assess the methodological quality of SRs with NMAs focusing on JAK inhibitors in RA [13].

## 2. Materials and Methods

### 2.1. Literature Search Strategy

This systematic review was registered with the International Prospective Register of Systematic Reviews (PROSPERO; number CRD420261279961) and was conducted in accordance with the guidelines of the Cochrane Collaboration and reported according to the PRISMA 2020 (Preferred Reporting Items for Systematic Reviews and Meta-Analyses) statement [14,15]. A systematic literature search was performed in the PubMed database “http://www.ncbi.nlm.nih.gov/pubmed/ (accessed 23 June 2025)”, and Embase database “http://www.embase.com/ (accessed 23 June 2025)”, with the last update in June 2025. Both MeSH terms and free-text keywords were used, combining the concepts “JAK inhibitors”, “rheumatoid arthritis” and “network meta-analysis”. No filters were applied. The full search strategy is described in Appendix A, Table A1 and Table A2, Supplementary Material. Search results were imported and managed into Mendeley Reference Manager (version 2.139.0) and duplicates were identified and removed.

### 2.2. Inclusion and Exclusion Criteria

The eligibility criteria included SRs including a NMA, assessing the efficacy and/or safety of JAK inhibitors in the treatment of RA. Only studies published in English language were considered, given being the predominant language for dissemination of biomedical research. Narrative reviews, editorials, letters to the editor, and commentaries, SRs without NMA, duplicate publications or SRs focused exclusively on a single JAK inhibitor were not included.

### 2.3. Study Selection Process

The study selection was conducted in two stages: (1) screening of titles and abstracts and (2) full-text review of potentially eligible articles. The selection process was performed by three independent reviewers. Disagreements were resolved through discussion and consensus. Screening was conducted manually, with Mendeley used for reference management and duplicate removal.

### 2.4. Methodological Quality Assessment

The methodological quality of the included SRs was assessed using AMSTAR 2 tool [12]. AMSTAR 2 is composed of 16 items that evaluate the risk of bias of SRs, including both randomized controlled trials (RCT) and non-randomized trials. Each item is rated using a trichotomous approach: Yes—when all requirements of the criterion were met; Partial Yes—when only some requirements were met; No—when the criterion was not met. AMSTAR 2 provides an overall rating of confidence in the results of each included SR, based on the presence of flaws in critical and non-critical domains. The critical domains are the following: protocol registered before commencement of the review (item 2), adequacy of the literature search (item 4), justification for excluding individual studies (item 7), risk of bias from individual studies being included in the review (item 9), appropriateness of meta-analytical methods (item 11), consideration of risk of bias when interpreting the results of the review (item 13) and assessment of presence and likely impact of publication bias (item 15) [12].

The tool was applied independently by three reviewers, and discrepancies were resolved by consensus, in accordance with the AMSTAR 2 guidance document [12]. The detailed instrument and its main guidance are described in Appendix B, Supplementary Material.

### 2.5. Data Extraction and Analysis

Data extracted included the main methodologic characteristics (e.g., reference/year, orientation followed, bibliographic databases searched, type of outcomes assessed, protocol registration, quality assessment scale, type of statistical model, heterogeneity/inconsistency assessment) and compliance level for each AMSTAR 2 item. A descriptive analysis was performed, with results presented in tables. The results of the AMSTAR 2 assessment were visually presented using robvis, version 0.3.0. [16].

## 3. Results

### 3.1. Study Selection

**Figure 1:**
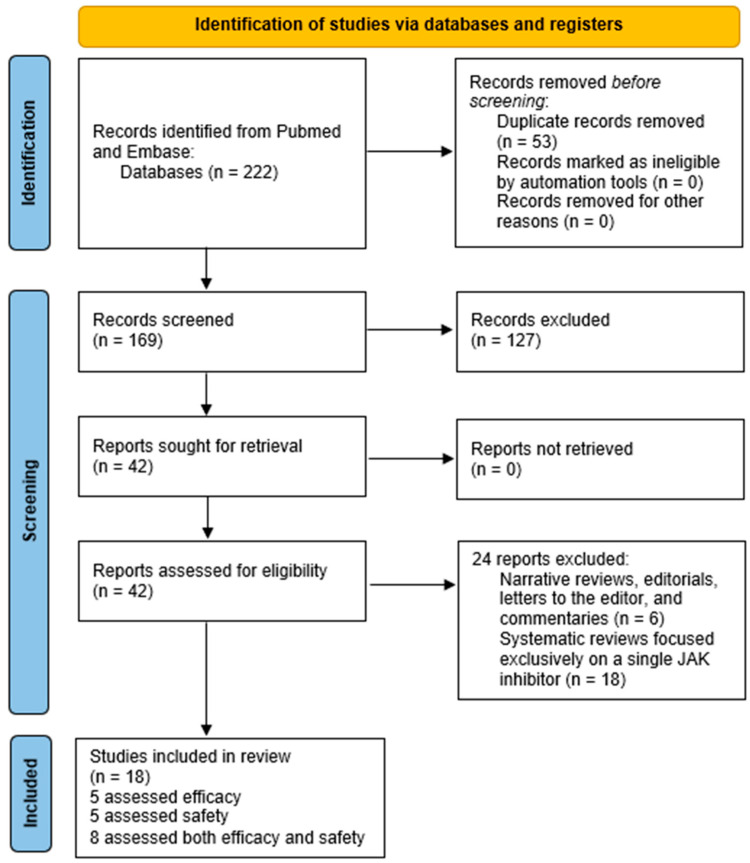
PRISMA flow diagram of the study selection process.

The literature search conducted in the PubMed and Embase databases yielded 222 potentially eligible publications. Following a rigorous application of the predefined eligibility criteria, a total of 18 SRs with NMA were included in the final sample [10,17,18,19,20,21,22,23,24,25,26,27,28,29,30,31,32,33]. Among these, 5 focused exclusively on the efficacy of JAK inhibitors, 5 on safety outcomes, and 8 assessed both aspects (Figure 1).

#### Characteristics of the Studies

In terms of methodological orientation, most reviews (n = 17; 94%) reported compliance with the PRISMA guidelines. Two of these also followed the Centre for Reviews and Dissemination (CRD) recommendations, and one explicitly adhered to the Cochrane Collaboration’ guidance. Only one review did not specify adherence to any formal methodological framework.

The bibliographic databases most frequently searched among the included SRs were the Cochrane Central Register of Controlled Trials (CENTRAL) (n = 18; 100%) and EMBASE (n = 17; 94%). Searches in PubMed (n = 10; 55%) and MEDLINE (n = 9; 50%) were performed in at least half of the SRs included. Overall, most studies conducted searches across at least three major databases, ensuring a comprehensive and systematic retrieval of relevant evidence. Regarding the type of outcomes assessed, the sample was evenly distributed: five reviews (28%) focused exclusively on efficacy, five (28%) on safety, and eight (44%) evaluated both efficacy and safety.

Concerning protocol registration, the majority of reviews did not report prior registration (n = 10; 56%). Among those who registered their reviews, PROSPERO was reported in six reviews (33%), the European Network of Centres for Pharmacoepidemiology and Pharmacovigilance in three reviews (18%), and the INPLASY in two reviews (11%). Some reviews reported registration in more than one registry or in different platforms. With respect to quality assessment of included primary studies, the most frequently used instruments were the Cochrane Risk of Bias 2 (RoB 2) tool (n = 10; 56%) and the Jadad score (n = 7; 39%), both used to assess clinical trials. Only one review (6%) did not report any quality assessment tool. No review applied more than one assessment method. The statistical modelling approaches were heterogeneous. The most common approach was a Bayesian fixed-effects model (n = 8; 44%), followed by a Bayesian random-effects model (n = 7; 39%) and frequentist random-effects models (n = 4; 28%). Only two reviews addressed more than one modeling approach (i.e., a Bayesian primary analysis with a frequentist sensitivity analysis). Finally, methods for assessing network heterogeneity and inconsistency varied across reviews. The most frequently reported approaches were the use of inconsistency plots or comparisons between fixed and random effects models (n = 6; 33%) and the node splitting (n = 6; 33%). In addition, there were reviews using other statistical methods such as the I^2^ statistic (n = 4, 22%), the Wald test (n = 3; 18%). Two reviews (11%) did not report any method for assessing heterogeneity or inconsistency. Several methods were often applied in the same review to assess robustness. Overall, these results indicate variability in methodological choices and reporting practices across the included NMAs, notably the limited use of prospective protocol registration and the heterogeneous application of quality-assessment instruments and inconsistency/heterogeneity assessment. The methodological characteristics of the eighteen SRs with NMAs included in this study are summarized in Table 1.

### 3.2. Methodological Quality Assessment (AMSTAR 2): Overall Compliance with AMSTAR 2

**Figure 2:**
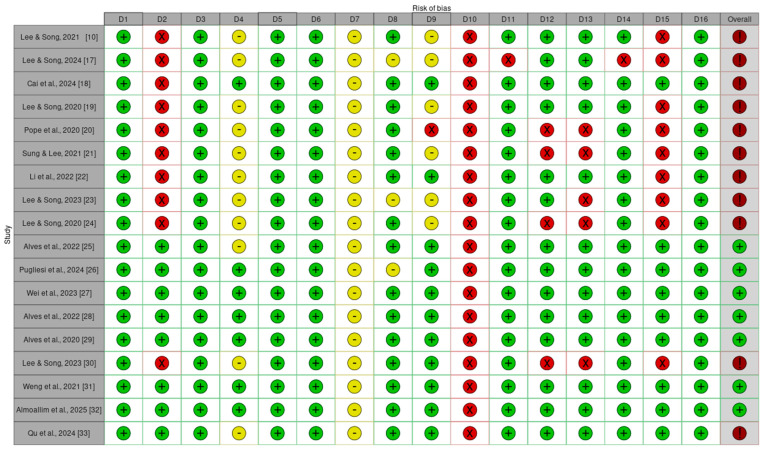
Methodological quality assessment for each SR with NMA, according to the AMSTAR 2 scale. Green (low concern; + signal) indicates “Yes” (all criterion requirements met), yellow (some concerns; - signal) indicates “Partial Yes” (some requirements met), and red (high concern; × signal) indicates “No” (criterion not met). Overall confidence rating is determined by the presence and number of critical and non-critical flaws, and can be interpreted as the following: Green (high; + signal; the SR provides an accurate and comprehensive summary of the results); Red (critically low; ! signal; the SR should not be relied on to provide an accurate and comprehensive summary of the available studies).

Compliance with AMSTAR 2 domains across the 18 included NMAs was heterogeneous, as illustrated in Figure 2. Several domains demonstrated consistently high adherence, while others revealed substantial methodological weaknesses.

The domains most frequently fulfilled were those related to structural components of the reviews, including clear definition of the research question (Domain 1), justification for study design (Domain 3), study selection performed in duplicate (Domain 5), duplicate data extraction (Domain 6), and reporting of conflicts of interest (Domain 16). All of these were fulfilled by 100% of the reviews. High compliance was also observed for the use of appropriate statistical methods (Domain 11) and for the discussion of heterogeneity (Domain 14), each reported by 94% of the reviews.

In contrast, the domains most closely associated with transparency and reproducibility exhibited the poorest performance. None of the reviews justified the exclusion of studies during the full-text screening stage (Domain 7; 0%), and none reported the funding sources of the included primary studies (Domain 10; 0%). Only 44% of the reviews presented a preregistered protocol (Domain 2), and just 39% described their search strategy in sufficient detail (Domain 4). Furthermore, only half of the reviews assessed publication bias (Domain 15; 50%). These patterns reveal notable methodological weaknesses in several domains considered critical to the reliability of SRs’ findings.

Beyond individual domain performance, all included reviews exhibited at least one critical methodological flaw, which affects the level of confidence that can be placed in their conclusions. The absence of protocol registration, incomplete reporting of search strategies, inconsistent assessment of publication bias, and lack of justification for excluded studies collectively represent gaps that undermine transparency and traceability, two pillars of SR integrity. Despite the strong compliance observed in structural aspects such as duplicate processes and conflict-of-interest reporting, the deficits in these critical domains weaken the overall credibility of the evidence generated. This is particularly concerning given the increasing reliance on NMAs to inform therapeutic guidelines and regulatory frameworks. As a result, unresolved methodological shortcomings may compromise both internal validity and the applicability of NMA findings in clinical and policy settings.

### 3.3. Comparative Analysis of Review Categories: Efficacy, Safety, and Combined Outcomes

**Figure 3:**
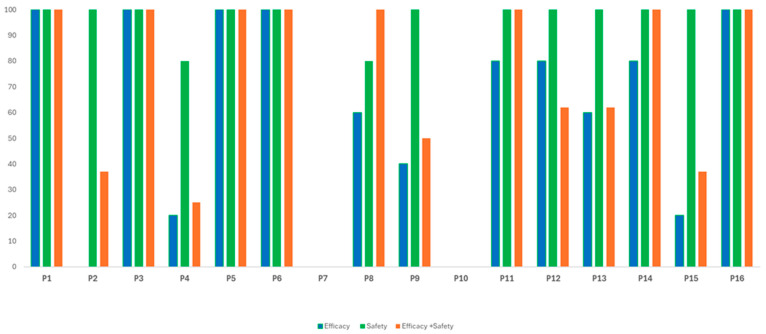
Comparative compliance with AMSTAR 2 domains by review focus. Bars represent the percentage of reviews in each category (Efficacy, Safety, Efficacy + Safety) that fulfilled each AMSTAR 2 domain (P1–P16 correspond to Domains D1–D16). Higher bars indicate greater compliance with the respective domain.

When stratifying the results according to the primary focus of the reviews, safety-focused reviews consistently demonstrated higher methodological quality compared to efficacy-focused ones, as shown in Figure 3.

This difference was particularly marked in several critical AMSTAR 2 domains. In Domain 2 (protocol registration), all safety reviews reported a pre-registered protocol (100%), while none of the efficacy reviews did so, reflecting greater transparency and methodological planning in the former group.

Similarly, in Domain 4 (comprehensive search strategy), 80% of safety reviews provided a well-detailed and appropriate search, compared to only 20% of efficacy reviews. In Domain 9 (risk of bias assessment), compliance was universal in safety reviews (100%) but observed in less than half of the efficacy group (40%).

The same pattern was found in Domain 15 (publication bias), with all safety reviews assessing this domain against only 20% in efficacy.

Transparency gaps were evident across both groups, particularly in Domain 10 (reporting of funding sources), which none of the reviews addressed, and in Domain 7 (justification for study exclusions), which was consistently absent. These findings highlight the systematic methodological advantages of safety-focused reviews, while also exposing shortcomings that remain common to both domains of investigation.

Reviews that assessed both efficacy and safety demonstrated an intermediate and often inconsistent methodological profile.

Although they performed well in structural domains, such as clearly defining the PICO question and conducting duplicate data extraction, they showed notable shortcomings in several critical areas. Only 37% reported a preregistered protocol (Domain 2), 25% described their search strategy in sufficient detail (Domain 4), and 37% assessed the risk of publication bias (Domain 15).

This variability likely reflects the additional complexity of synthesizing two distinct categories of outcomes, which may increase methodological demands and the likelihood of omissions. Consequently, although mixed reviews have the potential to provide a broader and more integrated perspective, their conclusions must be interpreted cautiously due to the heterogeneity observed in methodological quality.

## 4. Discussion

This study examined the methodological quality of 18 SRs with NMAs that evaluated JAK inhibitors for the treatment of RA, applying the AMSTAR 2 tool. Clear differences emerged across the reviews according to their primary focus: efficacy, safety, or both outcomes simultaneously. Safety-focused reviews demonstrated the strongest methodological performance. All reported a preregistered protocol, adequately evaluated the risk of bias in the included primary studies, and assessed the possibility of publication bias. This pattern likely reflects the heightened scientific and regulatory scrutiny typically associated with safety research, particularly in fields concerned with pharmacovigilance and drug-related harms. Similar findings have been described in other clinical domains, where safety-oriented reviews tend to perform better in critical methodological domains [13]. By contrast, reviews addressing efficacy presented considerable weaknesses. None reported a pre-registered protocol, and several described their search strategies only partially or with insufficient detail. Furthermore, the assessment of publication bias was also infrequent. These limitations have also been documented in earlier studies, which identified the absence of protocol registration and incomplete bias assessment as recurring problems in SRs of pharmacological interventions [34].

Mixed reviews, those addressing both efficacy and safety, showed intermediate performance. Although they complied well with several structural domains, they exhibited deficiencies like those of efficacy-focused reviews, particularly regarding protocol registration, search strategy detail, and publication-bias assessment. This may be attributable to the added complexity of synthesizing two distinct categories of outcomes, which can increase methodological demands and the risk of inconsistencies [35].

Across all review types, two shortcomings stood out: none of the reviews justified the exclusion of studies after full-text screening, and none reported the funding sources of the included primary studies. These omissions have also been highlighted in reviews from other clinical areas, raise concerns regarding transparency and traceability, two core dimensions of methodological quality [36].

It is important to note, however, that the present findings do not fully align with all previous literature. In a cross-sectional analysis of 127 safety-focused SRs in the surgical field, Zhou and colleagues observed that none achieved a high methodological quality rating, with the majority being classified as low or critically low quality [37]. This contrast suggests that the relative strength of safety-focused reviews observed in our study may not be universal, but rather dependent on the context and the scientific standards prevailing in a given field.

The implications of these results extend directly to clinical practice and regulatory decision making. NMAs are frequently relied upon as a basis for therapeutic guidelines and policy recommendations. Methodological flaws in critical domains, such as the absence of a protocol, insufficient assessment of bias, or failure to consider publication bias, may undermine the validity of the evidence and increase the risk of misleading conclusions [36,38]. In the case of JAK inhibitors, the impact of such flaws is particularly concerning, given the therapeutic importance of these drugs and the safety issues that accompany their use.

This study also has limitations. Although the search was expanded to include both PubMed and Embase, other databases may include relevant reviews not captured here. The assessment of methodological quality relied exclusively on AMSTAR 2, which, although widely validated, does not capture every possible dimension of review quality [34]. Finally, the analysis reflects the evidence available up to the date of the search, and more recent reviews or updated versions may not have been included.

### Future Implications

Future evidence syntheses on JAK inhibitors in rheumatoid arthritis would benefit from greater standardization and methodological rigor, particularly as NMAs are increasingly used to inform comparative decision-making [38]. Several improvements should therefore be prioritized. Protocols should be pre-registered in platforms such as PROSPERO (or an equivalent registry), enhancing transparency and reducing the likelihood of unplanned methodological changes. Literature searches should be comprehensive and fully documented (including reproducible strategies and clear justification of exclusions), and reporting should align more consistently with PRISMA guidance to facilitate reproducibility and interpretation of network structure and study contributions.

In addition, NMA-specific assumptions, like transitivity and consistency, should be explicitly assessed and transparently reported, alongside heterogeneity and small-study effects/publication bias where feasible [34,38]. Risk-of-bias and publication-bias assessments should be conducted systematically and integrated into interpretation, with consideration of structured approaches to rate certainty of evidence in NMA (e.g., GRADE for NMA). Finally, when reviews aim to evaluate both efficacy and safety, tailored methodological strategies are needed to address the additional complexity without compromising rigor; given evolving safety signals and regulatory recommendations for JAK inhibitors, living or regularly updated reviews, stronger long-term follow-up, and more consistent reporting of patient-centred outcomes would further strengthen the clinical and policy relevance of future NMAs [39].

## 5. Conclusions

In conclusion, these results reveal significant methodological discrepancies across efficacy-, safety-, and mixed-focused reviews of JAK inhibitors. These findings underscore the need for more consistent adoption of registered protocols, duplicate processes, and bias assessment. Addressing these shortcomings is essential if such reviews are to provide a reliable foundation for evidence synthesis, clinical practice and regulatory decision-making.

## Figures and Tables

**Table 1:** Methodologic characteristics of the SRs with NMAs included.

Reference	Guideline	Bibliographic Databases	Type of Outcomes Assessed	Protocol Registration	Quality Assessment Scale	Type of Statistical Model	Heterogeneity/Inconsistency Assessment
Lee & Song, 2021 [10]	PRISMA	MEDLINE, EMBASE, CENTRAL, ACR, EULAR	Efficacy & Safety	NR	Jadad scores	Bayesian fixed-effects model	Inconsistency plots, Fixed vs. random effects model comparison
Lee & Song, 2024 [17]	PRISMA	MEDLINE, EMBASE, CENTRAL, ACR, EULAR	Efficacy	NR	Jadad scores	Bayesian fixed-effects model	NR
Cai et al., 2024 [18]	NR	PubMed, EMBASE, Web of Science and CENTRAL	Efficacy	NR	RoB2	Random-effects model (Stata 14, RevMan 5.4)	Cochran’s Q test
Lee & Song, 2020 [19]	PRISMA	MEDLINE, EMBASE, CENTRAL, ACR, EULAR	Efficacy & Safety	NR	Jadad scores	Bayesian fixed-effects model	Inconsistency plots, Fixed vs. random effects model comparison
Pope et al., 2020 [20]	PRISMA	MEDLINE, EMBASE, CENTRAL	Efficacy	NR	NR	Bayesian random-effects model	NR
Sung & Lee, 2021 [21]	PRISMA	MEDLINE, EMBASE, CENTRAL, ACR, EULAR	Efficacy & Safety	NR	Jadad scores	Bayesian fixed-effects model	Inconsistency plots, Fixed vs. random effects model comparison
Li et al., 2022 [22]	PRISMA	PubMed, MEDLINE, EMBASE, CENTRAL	Efficacy	NR	RoB2	Bayesian random-effects model	I^2^ statistic
Lee & Song, 2023 [23]	PRISMA	MEDLINE, EMBASE, CENTRAL, ACR, EULAR	Efficacy	NR	Jadad scores	Bayesian fixed-effects model	Inconsistency plots, Fixed vs. random effects model comparison
Lee & Song, 2020 [24]	PRISMA	MEDLINE, EMBASE, CENTRAL, ACR, EULAR	Efficacy & Safety	NR	Jadad scores	Bayesian fixed-effects model	Inconsistency plots, Fixed vs. random effects model comparison
Alves et al., 2022 [25]	PRISMA; CRD	PubMed, EMBASE, CENTRAL, and ClinicalTrials.gov	Safety	PROSPERO, ENCePP	RoB2	Frequentist random-effects model	Wald test; Node-splitting; Loop-specific approach;
Pugliesi et al., 2024 [26]	PRISMA	MEDLINE (PubMed), CENTRAL, EMBASE, Web of Science, Scopus, LILACS	Safety	PROSPERO	RoB2	Bayesian random-effects model (MCMC)	I^2^ statistic, node-splitting
Wei et al., 2023 [27]	PRISMA, Cochrane	PubMed, EMBASE, CENTRAL	Safety	PROSPERO	RoB2	Frequentist random-effects model; Bayesian random-effects model	Cochran’s Q test, node-splitting analysis
Alves et al., 2022 [28]	PRISMA	PubMed, EMBASE, CENTRAL, and ClinicalTrials.gov	Safety	PROSPERO, ENCePP	RoB2	Frequentist random-effects model	Wald test; Node-splitting; Loop-specific
Alves et al., 2020 [29]	PRISMA-NMA, CRD	PubMed, EMBASE, CENTRAL, and ClinicalTrials.gov	Safety	ENCePP	RoB2	Frequentist random-effects model	Wald test; Node-splitting;
Lee & Song, 2023 [30]	PRISMA	MEDLINE, EMBASE, CENTRAL, ACR, EULAR	Efficacy & Safety	NR	Jadad scores	Bayesian fixed-effects model	Inconsistency plots, Fixed vs. random effects model comparison
Weng et al., 2021 [31]	PRISMA	PubMed, EMBASE and CENTRAL	Efficacy & Safety	PROSPERO, INPLASY	RoB2	Bayesian random-effects model	Node-splitting, design-by-treatment test, I^2^ statistic, τ^2^ and funnel plots
Almoallim et al., 2025 [32]	PRISMA	PubMed, CENTRAL, ClinicalTrials.gov, ICTRP Network	Efficacy & Safety	INPLASY	RoB2	Frequentist random-effects model	I^2^ statistic and τ^2^
Qu et al., 2024 [33]	PRISMA	CNKI, VIP, Wanfang, CBM, Pubmed, EMBASE, CENTRAL and Web of Science	Efficacy & Safety	PROSPERO	RoB2	Bayesian fixed-effects model and the Bayesian random-effects model	Funnel plots, Monte Carlo method and random-effects model

ACR, American College of Rheumatology; CENTRAL, Cochrane Central Register of Controlled Trials; CRD, Centre for Reviews and Dissemination; EULAR, European League against Rheumatism; ENCePP, The European Network of Centres for Pharmacoepidemiology & Pharmacovigilance; NR, Not reported; PRISMA, preferred reporting items for systematic reviews and meta-analyses; PRISMA-NMA, PRISMA extension statement for reporting systematic reviews incorporating network meta- analyses of healthcare interventions; RCT, randomized controlled trials.

## Data Availability

No new data were created or analyzed in this study.

## Supplementary Materials

The following supporting information can be downloaded at https://www.mdpi.com/article/10.3390/jcm15020725/s1, PRISMA 2020 Checklist.

## Appendix A. Search Strategy

**Table A1:** PubMed Search Strategy.

Search	Query
#11	Search: #4 AND #7 AND #10 Sort by: Most Recent
#10	Search: #8 OR #9 Sort by: Most Recent
#9	Search: “janus kinase inhibitor*” OR “JAK inhibitor*” OR “tofacitinib” OR “upadacitinib” OR “baricitinib” OR “filgocitinib” Sort by: Most Recent
#8	Search: “Janus Kinase Inhibitors” [Mesh] Sort by: Most Recent
#7	Search: #5 OR #6 Sort by: Most Recent
#6	Search: “network meta-analysis” OR “mixed treatment comparison” OR “multiple treatment comparison” Sort by: Most Recent
#5	Search: “Network Meta-Analysis as Topic” [Mesh] OR “Network Meta-Analysis” [Publication Type] Sort by: Most Recent
#4	Search: #1 OR #2 OR #3 Sort by: Most Recent
#3	Search: “arthritis, rheumatoid” Sort by: Most Recent
#2	Search: “rheumatoid arthritis” Sort by: Most Recent
#1	Search: rheumatoid arthritis [MeSH Terms] Sort by: Most Recent

**Table A2:** Embase Search Strategy.

Search	Query
#13	#4 AND #8 AND #12
#12	#9 OR #10 OR #11
#11	tofacitinib OR upadacitinib OR baricitinib OR filgocitinib
#10	jak AND inhibitor
#9	‘janus kinase inhibitor’
#8	#5 OR #6 OR #7
#7	‘mixed treatment comparison’
#6	‘network meta-analysis’
#5	‘network meta-analysis’ (topic)’
#4	#1 OR #2 OR #3
#3	‘rheumatoid arthritis’/exp
#2	‘rheumatoid arthritis’
#1	‘rheumatoid arthritis’/exp OR ‘rheumatoid arthritis’

## Appendix B. AMSTAR 2 Instrument Methodology

Domains:

(1) Did the research questions and inclusion criteria for the review include the components of PICO (Population, Intervention, Comparator, Outcome)?
(2) Did the report of the review contain an explicit statement that the review methods were established prior to the conduct of the review and did the report justify any significant deviations from the protocol? (critical)
(3) Did the review authors explain their selection of the study designs for inclusion in the review?
(4) Did the review authors use a comprehensive literature search strategy? (critical)
(5) Did the review authors perform study selection in duplicate?
(6) Did the review authors perform data extraction in duplicate?
(7) Did the review authors provide a list of excluded studies and justify the exclusions? (critical)
(8) Did the review authors describe the included studies in adequate detail?
(9) Did the review authors use a satisfactory technique for assessing the risk of bias (RoB) in individual studies that were included in the review? (critical)
(10) Did the review authors report on the sources of funding for the studies included in the review?
(11) If meta-analysis was performed, did the review authors use appropriate methods for statistical combination of results? (critical)
(12) If meta-analysis was performed, did the review authors assess the potential impact of risk of bias (RoB) in individual studies on the results of the meta-analysis or other evidence synthesis?
(13) Did the review authors account for RoB in primary studies when interpreting/discussing the results of the review? (critical)
(14) Did the review authors provide a satisfactory explanation for, and discussion of, any heterogeneity observed in the results of the review?
(15) If they performed quantitative synthesis did the review authors carry out an adequate investigation of publication bias (small study bias) and discuss its likely impact on the results of the review? (critical)
(16) Did the review authors report any potential sources of conflict of interest, including any funding they received for conducting the review?

The overall confidence can be interpreted as the following:

- High (zero or one non-critical weakness: the systematic review provides an accurate and comprehensive summary of the results of the available studies that address the question of interest);
- Moderate (more than one non-critical weakness *: the systematic review has more than one weakness, but no critical flaws. It may provide an accurate summary of the results of the available studies that were included in the review);
- Low (one critical flaw with or without non-critical weaknesses: the review has a critical flaw and may not provide an accurate and comprehensive summary of the available studies that address the question of interest);
- Critically low (more than one critical flaw with or without non-critical weaknesses: the review has more than one critical flaw and should not be relied on to provide an accurate and comprehensive summary of the available studies).

* Multiple non-critical weaknesses may diminish confidence in the review and it may be appropriate to move the overall appraisal down from moderate to low confidence.
